# Optimising Medication Use along Dementia Progression: Recommendations from a Qualitative Study

**DOI:** 10.3390/healthcare9080982

**Published:** 2021-08-02

**Authors:** Dalal Alsaeed, Mine Orlu, Felicity Smith

**Affiliations:** 1 Dasman Diabetes Institute, Clinical Care Research and Trials, P.O.Box 1180, Dasman, Kuwait City 15462, Kuwait; 2 School of Pharmacy, University College London, 29-39 Brunswick Square, London WC1N 51AX, UK; f.j.smith@ucl.ac.uk

**Keywords:** optimising medication use, dementia, caregivers, shared decision making, challenges, qualitative

## Abstract

Medicines management is known to be an integral part of the role of family caregivers; it also contributes to the burden and stress of caregivers’ experience. As dementia progresses, new challenges arise as a consequence, which negatively affects the ability of people living with dementia (PLWD) regarding practical decision making and may lead to a change of setting. The aim of this study is to identify and explore changes in medicines management and associated caregiver burden as dementia progresses. To examine medicines management and related issues across severities, a qualitative approach utilising face-to-face and telephone interviews with PLWD and their family caregivers in both the community and care-home setting in London was used. Follow-up interviews with family caregivers were also conducted to gain additional insight into change over time. Eleven family caregivers, ten PLWD, and eight care-home staff were interviewed in 2016. Findings identified how key changes along dementia progression affect medication use. These include changes to caregiver burden, respecting the PLWD’s autonomy and decline in capacity, scheduling and administration, choice of formulation, interactions with and between providers, and information needs. The findings assist in informing recommendations to optimise medication use and alleviate caregiver burden.

## 1. Introduction

There are approximately 850,000 people living with dementia (PLWD) in the UK, and these numbers are predicted to increase to 2 million by 2051 [1]. It is estimated that two-thirds of these PLWD live in their own home, while the other third reside in care homes [2,3]. As the number of PLWD increases, so does the strain on healthcare services to meet their needs. Dementia is costing the UK around £26.3 billion, with £11.6 billion attributed to family caregiver costs [1]. In England, the National Health Service (NHS) long-term plan was proposed and published in 2019; this was based on the policy foundation of the NHS five-year forward view highlighting the need to integrate care to meet the needs of a changing population [4,5]. Primary Care Networks (PCNs) were formed as part of the NHS long-term plan, with each network comprising general practices and other local health and social care workers to provide an integrated multidisciplinary approach within a specific geographical location. This includes a pro-active approach with healthcare professional teams on hand, enabling people to live independently at home for longer, supporting people to age well and improving recognition of caregivers and the support they need. Structured medication reviews (SMRs) and medicines optimisation services were officially implemented from October 2020 under the PCN Contract-Directed Enhanced Services (DES) [6]. These target specific patient groups in the community with medication-related needs, including those residing within care homes.

As PLWD gradually lose their ability to manage their medicines appropriately, family members or friends often step in to assist them. A family caregiver is defined as a relative or friend who has a personal relationship with the PLWD and provides assistance with a range of activities. Some of the medication-related activities include administering medicines, managing side effects, and maintaining the medicinal supply [7]. For PLWD, the ability to make decisions is often compromised. Some PLWD, in their desire to manage their own medicines, overestimate their abilities, as they would like to retain their autonomy [8,9]. Respecting autonomy (defined as “a person’s ability and opportunity to make decisions relating to his/her own wishes” [10]) as well as the need to take fuller responsibility for medicines can become a further challenge for caregivers [11,12].

Medication-related problems that family caregivers experience include practical issues in maintaining supplies, problems in administration of different formulations for both caregiver (e.g., managing packaging) and PLWD (onset of dysphagia), frequency of dosing of complex regimens, lack of information or understanding regarding dosing regimens, and concerns regarding effectiveness or adverse effects of medicines [7]. Decisions regarding medication for use when required, such as pain relief, may not be simple to administer due to the PLWDs’ inability to verbalise need for medication or express pain. These issues may lead to non-adherence to treatment regimens, sub-optimal clinical outcomes, and treatment failures, impacting the health status and need for hospital care [13].

Caregivers of PLWD are often older themselves and may have conditions for which they take medicines. They may have visual, dexterity, or other problems that may affect ease with which they can manage and administer medications for themselves and PLWD.

As dementia progresses, and family caregivers are no longer able to care for the PLWD at home, their care often shifts from their own homes to care homes [14]. Care-home staff are known to experience difficulties when administering medicines to people with dementia [15,16,17]. As a consequence of disease progression, possible changes in goals of care and the systems regarding medication management in an institutional setting as well as new issues in ensuring optimal use of medicines to achieve best treatment outcomes for PLWD will inevitably present themselves [18].

The progression of dementia is variable; all individuals have their own personal situations and health care needs, including medication. However, as the disease progresses, medication management may present many common experiences and challenges for PLWD and their caregivers. Identifying and understanding the medicines-related issues and problems that arise for PLWD and their caregivers as disease progresses is important for maintaining optimal use of medication and treatment outcomes and to address caregiver burden.

The aim of this study was to identify and examine medication-related problems that arise for PLWD and their caregivers as the disease progresses. This will inform the development of guidance for caregivers and healthcare professionals to ensure that important issues and problems are addressed in a timely way. Specific objectives were to examine medication-related problems from the perspectives of PLWD in the community, their family caregivers, and carers in care homes to encompass issues across dementia severities and settings and, in addition, to make recommendations as to how healthcare professionals may more effectively anticipate, identify, and address problems to achieve optimal outcomes for PLWD and reduce caregiver burden.

## 2. Materials and Methods

### 2.1. Study Design and Setting

This qualitative study was undertaken with family caregivers and PLWD living in their own homes and care-home staff from the Greater London area. Recruiting from both community and care-home settings was done to obtain data regarding the challenges of medicines management from across the disease severity spectrum and in the context in which care is provided. In the community sample, PLWD (likely to be less advanced in their disease) as well as their caregivers were invited to take part, as recognising partnerships between these caregivers and PLWD in the management of medicines may be important. In addition, data were also gathered from family caregivers at two timepoints to provide an opportunity to examine the emergence and resolution of new problems over time.

### 2.2. Sampling and Recruitment

In the community setting, PLWD and their family caregivers were recruited through the Alzheimer’s Society. A purposive sampling approach was employed. Recruitment was conducted through dementia advisors and attendance at Dementia Cafés. Three London boroughs were chosen taking into account geographical area, prevalence of dementia, and socioeconomic and ethnic diversity.

The eligibility criteria for PLWD were: having a diagnosis of mild cognitive impairment or dementia and living in their own home. Family caregivers were 18 years and older, providing assistance with medication management for a PLWD. All participants needed to be able to converse in English and be willing and able to give informed consent for participation.

Care homes were identified through the Care Quality Commission (CQC) website and were purposively selected to include a varied sample of homes caring for residents with dementia, providing different types of care, and representing different home sizes. Managers of eligible care homes (registered as a nursing or residential care home with the CQC and caring for residents with dementia) were contacted and, if consented, were included in the study. Individual staff members were subsequently approached and invited to take part.

As is common in qualitative studies, sample size was governed by principles of sampling to saturation, i.e., until no new themes and issues were emerging [19]. For both community and care-home data sets, iterative analysis was undertaken to ensure data saturation was reached.

### 2.3. Ethics

Ethical approval was provided by the National Research Ethics Service (NRES) Committee South East Coast-Surrey Research Ethics Committee on March 2015 (15/LO/0177).

### 2.4. Data Collection

Data were collected in face-to-face, semi-structured interviews except for the follow-up interviews with family carers in the community, which were conducted by telephone. Three sets of topic guides were developed for PLWD, their family caregivers, and care-home staff. The topic guides were informed by existent literature and preliminary discussions with representatives of family caregivers and care-home staff, including two patient and public involvement meetings convened by the research team. This ensured that domains and topics relevant to the study objectives were included. The topic guide was piloted with three former family caregivers who helped improve the questions. They also reviewed information leaflets and other project documentation. The interview schedule gathered views and experiences of caregivers and PLWD regarding activities and problems in organisation, scheduling of medicines and interface with formal care, medicines use and administration at home, knowledge and information, partnerships between caregivers and PLWD, caregiver burden, and personal characteristics of participants. Caregivers provided an approximation of dementia severity as well as stated how long they can leave the PLWD on their own; this was utilised in previous studies [20,21].

PLWD and their family caregivers were interviewed in their homes; follow-up interviews were done via telephone. Care-home staff were interviewed in their place of work. Data collection continued until data saturation.

### 2.5. Data Management and Analysis

The interviews were audio-recorded and transcribed verbatim. Principles of thematic framework analysis were used [22]. This utilises both a deductive and an inductive aspect to analysis; the initial thematic framework was informed by the study objectives and domains of the interview guide as well as a review of existing literature [7]. Combining this with an inductive approach enabled emergence of new themes and sub-themes within the framework to achieve the study objectives. Constant comparison of data items was employed to explore differences and similarities in perspectives and contexts. Community and care-home data sets were coded and analysed separately in the first instance and then brought together to explore experiences and problems related to each of the themes and as disease progressed; community follow-up interviews were analysed alongside baseline interviews. To ensure validity and reliability, for a sample of transcripts from each data set, all steps in the development and application of the coding frame were undertaken independently by three members of the research team; any discrepancies were discussed to reach a consensus. The indexing process was systematically applied with the updated coding frame on all the data sets. The textual data was synthesised and the codes refined continuously. Patterns across the data were identified, and concepts were generated to create a conceptual model illustrating areas requiring optimisation with medication use. The data-management software NVivo 11 (QSR International Pty Ltd. Version 11, 2015) was used to facilitate coding and retrieval of codes.

## 3. Results

### 3.1. Characteristics of Participants

Nine dyads (PLWD and their family caregiver), two family caregivers, and one PLWD were interviewed from the community setting. Characteristics of the sample are reported in Table 1. Eight staff members from four care homes around London participated (Table 2). Ten family caregivers and one PLWD agreed to the follow-up interview via telephone.

Synthesising all three sources of data, this paper reports the findings across care settings and in relation to the progression of dementia and how to optimise medication use along this trajectory. The themes that emerged include: (1) caregiver burden, (2) interaction between staff and residents, (3) PLWD autonomy and capacity, (4) scheduling and administration of medications, (5) choice of formulation, (6) interactions with and between providers, and (7) information and knowledge. The themes and sub-themes can be found summarised in Table 3.

### 3.2. Caregiver Burden

According to family caregivers who are caring for PLWD at home (from early to moderate disease), as the PLWD’s cognition and capabilities decline, more responsibilities are gradually assumed by them, which may in turn *increase their burden*. Their burden is also affected by their *expectations for the future* regarding the PLWD’s decline in cognition and concern about meeting future medication needs. This is more so for spouses who are elderly themselves.

*“Yeah, at the moment he’s not at that stage, you know, at the moment he just happily just takes them when I give them to him. But I should think further down the line that could be a problem if he needs to swallow them.”*

Caregiver 8, 70 years

For some caregivers and PLWD, minimal changes may occur regarding their respective roles on this trajectory. Nevertheless, both the PLWD and their family caregiver are getting older, which may make their role more difficult to manage.

*“I think whether it’s just me getting older and more crotchety (laughs) or whether illness is developing and I think things are, it’s not medication that’s becoming more difficult, but I think life generally is.”*

Caregiver 5, 89 years

Progression of dementia may indicate a *change in support needs*. Even though the initial diagnosis of dementia is challenging to adjust to, many of the participants did not express need for support. With the progression of dementia and worsening of symptoms, such as inability to communicate well and development of challenging behavior, caregivers felt overwhelmed and required more support, especially as their own health deteriorates.

*“No, while my memory holds out, all’s well. But of course if that starts to go, then we would need a lot more support.”*

Caregiver 6, 72 years

In the care-home setting, it was evident from observations and interviews how distractions and resident behaviour prolong medication rounds, which in turn aggravates staff burden. Staff experience stress and fear that these distractions lead to medication errors.

*“Umm, really I am having about 10 to 12 years’ experience in this country. I work in nursing home quite a lot and people with dementia it’s, really it’s hard to give the medications, in that one stage it is very, very difficult, you know.”*

N1, female, 55 years

Alleviating care-home staff burden may enable them to remain in their position for longer, thereby maintaining staff levels and reducing staff turn-over.

### 3.3. Interaction between Staff and Residents

A positive relationship between staff and residents may make medication administration relatively easier. Furthermore, staff who are familiar with individual residents are in a better position to identify, monitor, and report problems to HCPs as well as liaise between HCPs and residents. Not having trust or familiarity may make the resident question the medications and make administering medications a long process.

*“They get agitated, especially when they don’t know you, they don’t recognise your face. And you have to find a way to give it to them or just to come back and give it later. You have to adapt yourself to them, you know? And that’s the most difficult thing, sometimes when they get agitated and they don’t know you. You always ask for help with the permanent staff (…)”*

N2, female, 26 years

Progression of dementia may also coincide with the *development of behavioural changes* and *willingness to take medications*, thus affecting clinical outcomes. Refusing medications may also relate to the trust between the PLWD and the person administering the medications and their approach. Challenging behaviour may sometimes relate to preferences to medications, so an alternative formulation may be prescribed. This is balanced against the *preservation of the PLWDs’ autonomy* and respect of their wishes.

*“Well like with Mrs. W., he’s written a letter saying if we can’t get her medication down, the GP’s aware of it, so if we can’t, we can’t, there’s no more, nothing else they can really do.”*

C4, female, 47 years

### 3.4. PLWD Autonomy and Capacity

*Decline in speech and communication abilities* manifests along dementia progression, which consequently may result in a decreased ability to verbalise pain and need for medication and to communicate side effects. This may increase caregiver concern, as they are unsure when to start or stop a medication.

*“Anyway, I started on the tablets yesterday, one a day, but I was reading the leaflet this morning, quite, quite worrying. And what is very worrying is that in cases like (her husband), he wouldn’t be able to say to me ‘oh I’ve got ringing in my ear’ or ‘I’ve got bad stomachache’ or ‘I’m feeling dizzy’. People with dementia wouldn’t be able to tell you that.”*

Caregiver 1, 74 years

This decline in speech and language also affects how medications are given in care homes, as staff may find it confusing not knowing if the PLWD is willing to take the medication.

*“Some of them, no means yes, and when they say yes, [it] means they say no, ‘I said I told you no!’ I say ‘no, you told me yes, you’re ready to take’ they say ‘no’. So it’s very challenging, but we keep going, we try to make them...more comfortable and happy.”*

N3, male, 48 years

Decline in cognition may have a negative effect on the *PLWD’s autonomy, capacity, and decision-making abilities*. In many cases, there is a decrease in independence and a subsequent increase in reliance on the caregiver. Medication-related activities and decision making are gradually assumed by the caregiver. Decision making is a complex process linked with the PLWD’s autonomy and wellbeing and the caregivers’ knowledge and health literacy. Caregivers were seen struggling to strike a balance between their knowledge and responsibility and the PLWD’s autonomy. This compromise in the PLWD’s autonomy affects how medications are managed and administered.

PLWD who were at the moderate to severe stage required supervision when they were given their medication, which compromises their autonomy in this context. Practical problems with medication use and behaviour issues may also evolve, which raise ethical concerns for caregivers. Some caregivers may have to adopt strategies to manage and administer medications in a way that still respects the PLWD’s autonomy. This was seen through involving them in the organisation and scheduling of medications at home, however minimally, and providing some control over the administration of medications. This was also seen in some care homes where staff took steps to respect autonomy by taking consent and allowing minimal supervision.

### 3.5. Scheduling and Administration of Medications

The scheduling of medications forms a major component of the medication management process. Factors identified that affect this process include the dosing schedules and changes in medications. Dosing schedules where medications are only given in the morning and the evening were seen as optimal by most caregivers and PLWD, as they can be incorporated easily into their daily routine. A change in routine can also have an impact on scheduling medications; attending social events or having to go to appointments may cause medications to be missed.

*“But I’m kind of building that into the routine because when she was having lunch at home it was easy, I just kept the tablet next to her lunch plate. So now before I leave the house to take her to her lunch club, I have to go to the kitchen and check that I haven’t forgotten anything, then I give her her tablet before I leave. So it’s just as you say, building in cues whenever we want to remember.”*

Caregiver 1, 60 years

Change in medications, such as the addition of new ones or temporary ones (e.g., antibiotics), may be seen as a hassle to some caregivers, especially when the PLWD is already on a number of medications, and it may lead them to decide to omit a medication that they do not deem as important as others.

Caregivers have to sometimes individualise strategies for how to approach PLWD who display challenging behaviour. These can range from placating and coaxing the PLWD to finding a suitable time to give medications. Understanding the PLWD’s mood would greatly assist with approach, as seen also in the care-home setting. Talking to PLWD and explaining to them why they are taking medicines can be very helpful. It is not just the familiarity with the caregiver that is important but also what is said and how it is said.

As PLWD age and their health and cognition deteriorate, the *number of medications may increase, and regimens become more complex*. This contributes to caregiver and PLWD stress and PLWD willingness to take medication.

*“Sometimes you think it’s too many tablets or something. It’s just not easy.”*

Caregiver 7, 74 years

*How medications are managed and scheduled* (e.g., dosing intervals) at home may need to change as dementia progresses. Although multicompartment aids (MCAs) may sometimes be seen as a solution for someone with dementia to retain their ability to manage their medications at the early stages, it may not necessarily be beneficial for all PLWD, as at a certain point, they may become impractical. The assumption that blister packs will be easier for PLWD at home to distinguish and remember their medications should therefore be made with caution.

*“Before you see I don’t have to give her medication, she go buy medication by herself. But now if I tell her that (pointing at blister pack) she couldn’t tell what’s [with] lunch, what’s you know, she can’t! I have to supply her, I have to give her.”*

Caregiver 3, 87 years

In care homes, the organisation of medications (in original packaging versus MCA), distractions, and the number and type of formulations may all prolong the medication round and affect medication-administration timings. Staff administering medications may be able to prompt reviews to remove unnecessary medications. In addition, development of strategies to deal with distractions from residents and increasing staff awareness of the importance of running the round without disruptions may assist.

### 3.6. Choice of Formulation

The choice of formulation is important to ensure that medications are taken as prescribed and without problems. The characteristics of formulations, such as the type, size, shape, colour, taste, and quantity, need to be taken into account. For caregivers, formulation type was an important concern. This related to ease of use, ease of administration, swallowing ability, and need for multiple dosing and monitoring. Prescribing decisions should take into account formulation characteristics to ensure acceptability to both the caregiver and PLWD and to promote adherence. Packaging can also be troublesome; medications and their packaging should be taken into consideration by HCPs with both the PLWD and caregivers’ abilities in mind.

The development of *swallowing difficulties* may become more common at the moderate to severe stage, requiring more *appropriate formulations*. This means that certain formulations become more problematic, and people resort to medication modification. Some modifications were conducted without the required information from HCPs. Another consequence of cognitive decline in some participants was forgetting how to swallow, and thus, they can choke on medications or not be able to swallow them at all. Choice of formulations and route of administration require assessment of suitability as dementia progresses to ensure PLWD are receiving their treatment in a safe and effective manner and with dignity.

*“Now that we’re at a stage of her dementia where she maybe thinks of herself as being in her childhood a lot of the time...a lot of her mind is like the mind of herself as a child and therefore somebody putting an oestrogen pessary into you is quite distressing, it would be very distressing for a child. ‘What are they doing to me?’ it’s like abuse, it feels like abuse if you don’t understand.”*

Caregiver, 57 years

In care homes, choosing a suitable formulation that meets the residents’ preferences and abilities is imperative to ensure medications are taken as prescribed. Physical barriers, such as swallowing ability, eyesight, and manual dexterity, may pose problems with medication use and dictate a change to a suitable formulation. Formulations can also be switched according to residents’ preferences and behaviour. Inappropriate formulations have negative implications through unsuitable modifications that may affect the efficacy of the medication.

*“Umm, inhalers, yes, because due to the level of dementia that the, they don’t understand that you have to actually inhale (inhales) deeply, and so a lot of inhalers, such as the Ventolin, they don’t get the full use, even with a Volumatic.”*

C1, male, 52 years

### 3.7. Interaction with and between Providers

Caregivers and PLWD interact with formal care along a variety of points from diagnosis to obtaining medication to support and services. The relationship the caregiver and PLWD have with HCPs may have an impact on medication use. The factors associated with this relationship, such as trust in the HCP and effective communication, can make the dyad feel supported and comfortable to discuss issues they might have with their medications or voice any concerns. Inclusion of both the caregiver and PLWD in discussions with HCP, such as the general practitioner (GP) and pharmacist, is favourable, as it assists in creating a stronger relationship and may also ensure that the dyads’ concerns are addressed. The PLWD’s inclusion in the discussion also respects their autonomy.

*“I think she (the doctor) doesn’t like me! … She is not used to me, and I am not used to her. Umm…yeah…one doctor is…umm…listening to you, the other doctor is not listening to you, so…”*

PLWD 1, 74 years

Continuity of care and long-term relationship with the GP were important. Seeing the same GP helped foster communication and trust. GPs familiar with their medications were seen as helpful in prescribing decisions.

*“But we try and see the same chap, not always possible, but the one who knows us. Because I think it’s quite important that they know the kind of medication you’re on, and they know what you’re like.”*

Caregiver 2, 73 years

It was evident that staff relied heavily on the pharmacy and interacted with them frequently. The interactions were not always positive, which can make some staff disinclined to interact with the pharmacy. This may mean that pharmacists are not utilised for medicine information. Other participants would approach the pharmacist for help prior to the GP; this was attributed to trust, accessibility and a good relationship.

*“Yeah, he’s always there at a drop of a hat if we need him or we’re not sure. He comes and does inspections, he does, one of his staff does training with the staff and everything, he’s always there, sort of, he brings medication to us out of hours if we fax a prescription through, he’ll bring it in himself if he’s got no one to bring it in. He’s very good.”*

C4, female, 47 years

Care homes that ensure that the multi-disciplinary team (MDT) has regular meetings that include care-home staff may assist in making the medication-use process easier for staff. This is through better and regular communication of concerns within the team to ensure issues are dealt with quickly and appropriately by including all the relevant HCPs’ opinions.

### 3.8. Information and Knowledge

Caregivers may sometimes make decisions without the required knowledge, which may sometimes conflict with what is best for the PLWD’s health. This lack of information not only affects decisions but may also increase caregiver burden relating to medication use. Identifying areas where caregivers and PLWD lack information can assist in targeting these areas and ensuring caregivers and PLWD make informed decisions about medication use, thereby optimising medication use and decreasing caregiver burden associated with decision making. Areas where lack of information affected medication use were about appropriateness of formulations, modification of medications, medication interactions, side effects, and effectiveness and need for medications.

*“So what I have to do is sometimes give them with orange, and again, I find that a worry because I think, I know some medications you can’t take grapefruit with, so I’m thinking, does orange do the same sort of thing? But having said that, I think it’s more important that he has the tablets than worrying about the small amount of orange he’s drinking.”*

Caregiver 1, 74 years

The lack of knowledge concerning certain aspects of medication use and the questionable strategies utilised by some participants in care homes highlight areas requiring further education and training. Change in medications, such as interchangeability between brand and generic, may cause confusion to some staff when they receive the medications from the pharmacy. Regarding medication modification, although staff have weighed the benefits with the risks, the questionable methods they employ might affect medication effectiveness and/or cause adverse effects. One carer was observed mixing three different liquid medications to administer and was not aware that this is a form of modification and the legal and clinical implications involved. Staff have to monitor residents when giving medications and identify any problems that the residents have. This assists them in recognising issues with certain formulations, such as inability to swallow or disliking taste, and reporting to the GP.

*“But we, we, in a week, we noticed most of our residents wasn’t able to take it or chew it and find that it was spat out somewhere else, so again, approach the doctor, and now we’re back on the Adcal dissolvable now.”*

C2, male, 55 years

When care-home staff were asked if they thought residents who chewed medications might have swallowing difficulties, they were unsure. This uncertainty may indicate that care-home staff need training on how to identify patients with swallowing problems and report them for assessment.

Caregivers and PLWD were not always aware of services or support even though they needed them. Reasons include no signposting by HCPs or that they felt they were not at the stage where they required it yet. Health literacy is known to decline with age, and HCPs should acknowledge this and be able to meet these informational needs and signpost caregivers and PLWD and/or refer them to the appropriate source of information along disease progression.

## 4. Discussion

The findings provide a wider view on the spectrum of problems faced by PLWD and those who care for them at different stages of dementia severity; this included medication-specific issues (such as lack of appropriate drug formulations tailored for the administration-related needs of PLWD), changes to caregiver burden in relation to decline in PLWDs’ cognition, and respecting the PLWDs’ autonomy along the trajectory. Understanding the context of these problems enables the provision of tailored recommendations that may help optimise therapy for each setting, improve clinical outcomes, and reduce caregiver burden. Along dementia progression and the resultant changes to the medication use process, the findings have shown various similarities and differences between care settings. In many cases, what seems to be a difference may in reality be attributed to the care setting and environment, such as the availability of information and support from HCPs.

There is limited research concerning acceptability of formulations in older people in comparison with other age groups, especially PLWD [23]. It is imperative to achieve optimal medication use in PLWD by asking about how they feel about their medication and/or formulation [24]. Although the current findings did not test the acceptability of specific formulations, nevertheless, it still contributes to knowledge by identifying the importance of the choice of formulation (e.g., type of dosage form, route of administration) in this patient population and their caregivers.

Caregivers of PLWD are known to have more burden in comparison with other caregivers due to the symptoms of decline in cognition, which may include decline in communication and a change in behaviour in the PLWD [20]. Administration of medications may be a hassle experienced by caregivers within the medication-use process and contributes to their burden [25], and this was true in the present study as well. The caregiver’s increasing role with managing complex therapies, decision making with minimal information, and maintaining the PLWD’s autonomy may also add to their burden. These were previously explored but not in the context of medication use [26].

The importance of the PLWD’s autonomy within the medication-use process and endeavouring to respect it as cognition declines was identified. Overriding their autonomy may result in challenging behaviour and places more pressure on the caregiver. Respecting older people’s autonomy and providing them with dignity have been recognised as crucial in healthcare in the UK [27] and is imperative in person-centred care. Sharing medication-related responsibilities may be beneficial for strengthening the partnership between the caregiver and PLWD and may alleviate caregiver burden. Involving PLWD in their medication decisions and providing them with choices increase their feelings of worth, and caregivers are central in enabling this. The partnership may enable the sharing of medication-related activities and therefore preserve the care-recipients’ autonomy, seen in the current findings and previously [12]. Strategies worked best when they were tailored to their cognitive decline and abilities and respected the PLWD’s autonomy.

### 4.1. Practice and Policy Implications

With the implementation of the NHS long-term plan, these findings provide valuable insight and can be developed as a guide to support medicines optimisation in PLWD, also taking into account the perspectives of family caregivers. In addition, the findings also shed light into the challenges in care homes, for which the NHS long-term plan sets to enhance care within. Guidance from the NHS in September 2020 proposed the implementation of the new structured medication reviews (SMRs) and medication-optimisation guidance for PCNs [28]. The guidance emphasises implementation in care homes; this should also include PLWD and older people living in the community to ensure their needs are met. In addition, we propose that a caregiver should be included, with the shared decision-making goal in mind. The findings identify a need for a more individualised approach towards medication use for PLWD, which involves prescribing appropriate drug formulations and addressing barriers to effective use, which can be used in the implementation of SMRs. A recent study aimed to develop a medicines-management intervention for PLWD in the community by gaining the perspectives of GPs and pharmacists [29]. They concluded that a pharmacist-based intervention was more feasible than a GP-centred one; it was based on a medication-use review with an adherence check component that is delivered via an online video.

We propose the use of a tool informed by the current study findings to be used within the context of SMRs in GP consultations in the care-home setting or by a pharmacist. The consultation with the GP is an important setting where medication-use issues may be identified and addressed. According to the NHS, only 26% of PLWD (those with a coded diagnosis of dementia) in England had their medication reviewed by a GP in the preceding 12 months [30]. Ongoing input is necessary, and as such, continual contact with the PLWD and their caregiver along the progression of dementia is recommended, with a proactive approach in relation to progression of disease. Having a named GP for caregivers and PLWD may assist in achieving this continuity in care and ensure their familiarisation with their needs. Table 4 outlines the proposed questions as part of the tool.

The tool should be adapted depending on the care setting: whether in the community or in care homes. Below are some points to consider when implementing this proposed tool in the community:

- Consultation should include both the caregiver and PLWD to gather full scope of issues;
- Role of caregiver as an expert should be acknowledged;
- Confidentiality issues should be addressed at the start to ensure PLWD’s autonomy is respected;
- Content is dynamic and would need to change along dementia progression to adapt to changes in medication use and the PLWD and caregivers’ needs, such as facilitating transition in care in the early stages, assessing swallowing difficulties, and challenging behaviour in later stages;
- In the beginning, they should try to establish an informal arrangement between the caregiver and PLWD about the PLWD’s preferences for medications and formulations to anticipate future changes in treatment. This agreed medication plan should be updated along the trajectory and include:
  ○ Medications desired by the PLWD, such as prescribed or when needed (e.g., pain relief);
  ○ Preferences when taking medications, such as with/without food, to assist administration if required;
  ○ Preferences and acceptability of certain formulations (tablets, capsules, liquids, patches, creams, etc.);
- HCPs may not be recognising and assessing swallowing difficulties in older people [31]. Asking about behaviour when administering medications or issues with certain formulations may indicate the presence of swallowing difficulties and therefore require an assessment. In later consultations with further cognitive decline, the GP may assess swallowing difficulties and accordingly refer to a specialist; and
- Assessment of suitability of formulations and route of administration is necessary.

The NHS long-term plan also highlights optimising care in care homes. Previous research has demonstrated that PLWD in care homes are prescribed potentially inappropriate medications [32] and that the pharmacist and GP should be utilised in this care setting to optimise medication use as proposed in the SMR guidance [28]. The current findings support a multidisciplinary team in every care home; the team would also include a pharmacist to provide their expertise in reviewing medication issues and resolving them accordingly. Regular reviews of residents’ medications should be made in care homes, and the pharmacist is advocated to perform them.

The National Institute for Health and Care Excellence (NICE) published a full guideline on managing medications in care homes to provide recommendations for care [33]. They support the inclusion of the resident, their family caregiver, and a pharmacist in reviewing medications. Care-home staff should also acknowledge the resident’s right to refuse medications. Furthermore, staff should be aware of how to administer different formulations. These are all advocated by the study’s findings. Recommendations that have emerged from our findings include:

- Involving PLWD in decisions about changes or additions to their medication regimen and explaining why;
- Respecting their need for independence by providing subtle supervision of medication taking and following their preferences for how medications are administered (such as placing all the tablets in their hands or on the table for them to take one at a time);
- Providing them with choices of drinks to take with the medications; and
- Respecting their decision to take their medications at another time but still endeavouring to approach using friendly language when they refuse medications.

With the COVID-19 pandemic, medicines-use optimisation in this vulnerable population is even more crucial. With the multiple lockdowns and regulations in place as well as the risk of infection, various factors can emerge that may drastically affect medication use, such as changes in routine, caregiver health, reduced contact with GPs and pharmacists in the community, and the low priority placed on medication-use reviews [34]. Interventions targeting PLWD and their caregivers should be brought to the forefront to achieve their optimal health outcomes as their cognition and health declines and ensure they are taken care of at home for longer.

### 4.2. Limitations

The community sample may not be representative, as people from organisations, such as the Alzheimer’s Society in this case, may be motivated to be included in research. They are also from specific areas in London and may not be generalizable to other populations. However, the participants were from diverse socio-economic and ethnic backgrounds and therefore can be representative of a London population. The sample size in the present study may be considered small, but recruitment and data collection continued until data saturation was achieved [19]. Furthermore, unlike quantitative research, qualitative research yields a large amount of textual data to be analysed, and thus, small samples are sufficient to achieve the research aim and objectives [35]. The present study did not utilise a scale to directly measure disease severity, as these scales, such as the MMSE, may be seen as distressing by some PLWD and their caregivers [36]. Furthermore, recruiting from the Alzheimer’s Society guaranteed that participants had a form of cognitive impairment.

## 5. Conclusions

Medication use in PLWD is a complex process and is influenced by a variety of medication- and person-related factors. The study has identified challenges to medication use along the dementia trajectory across care settings, although these did not have a uniform presentation but are variable and may occur at any stage. These include caregiver burden, respecting the PLWD’s autonomy and decline in capacity, scheduling and administration of medication, formulation suitability, interactions with and between providers, and information needs. There is a need for ongoing input as dementia progresses to identify and address these changes as they occur, and the findings have enabled the perspectives of family caregivers, PLWD, and care-home staff to contribute to recommendations for prescribing and administration of medicines as well as to help anticipate, identify, and address issues that may optimise medication use to improve treatment outcomes and reduce caregiver burden though a holistic approach.

## Figures and Tables

**Table 1:** Characteristics of Caregivers and PLWD in the Community.

	PLWD (*n* = 10) *	Family Caregivers (*n* = 11) *
**Age (mean (range))**	80.6 (72–89)	65.8 (57–89)
**Gender (female)**	6	6
**Relationship to PLWD**		
Daughter	-	2
Spouse (wife)	-	4
Spouse (husband)	-	5
**Approximate severity of dementia**		
Mild	4	-
Moderate-severe	3	-
Severe	3	-

Note *: Participants interviewed consisted of 9 dyads, 1 PLWD, and 2 family caregivers.

**Table 2:** Characteristics of Care-Home Participants.

	Carers (*n* = 5)	Nurses (*n* = 3)
**Age (mean (range))**	44.4 (0–55)	43 (26–55)
**Gender (male)**	3	1
**Job Title**		
Residential care/unit manager	3	-
Senior carer	2	-
Nurse	-	3
**Years working**		
Less than 5	-	2
5–9	2	-
10–20	1	-
21–30	1	-
More than 30	1	1
**Type of care home**		
Residential	4	-
Mixed	1	-
Nursing	-	3

**Table 3:** Themes and Sub-Themes.

Theme	Community Setting	Care-Home Setting	Further Issues Regarding Progression
Caregiver burden	Own health needs→getting older Responsibility and decision making Increase in burden affects how well they can manage and administer medications Ultimately may cause PLWD to transition to care home	Staff experience stress in completing medication round on time and ensuring no medication errors occur Dealing with residents with dementia and behaviour changes difficult for staff to manage	Transition from self to caregiver-led care: Gradual assumption of medication-related activities by family caregiver Endeavour to respect PLWD autonomy with transition Caregiver expectations for the future: Deterioration in caregiver health and getting older may affect ability to manage medications Fear for how to cope when decline in cognition affects medication use
Interaction between staff and residents	_	Rapport and familiarity between staff and residents important when administering medications Medications not given on time and/or wasted due to challenging behaviour Medication round is longer, affecting other residents’ medications (due to challenging behaviour) Can result in undermining autonomy to administer medications	Behaviour changes: Need individualised approach with medication administration Quiet and familiar environment may become important for medication administration
PLWD autonomy and capacity	Wish to maintain autonomy by both PLWD and caregiver Strategies developed to respect autonomy with medication management and administration May lead to problems that may affect adherence May be linked with behaviour changes and willingness to take medications Maintaining autonomy may contribute to caregiver burden	Need individualised approach that respects autonomy Staff take steps to respect autonomy when administering and supervising medication taking May make residents more willing to take medications when they are given more independence Giving freedom may mean some medications missed	Decline in cognition and communication: Decline in understanding and recall Unable to recognise and take medications When and how medications are given is affected (e.g., PRN pain relief) Concerns about side effects may not be voiced PLWD Autonomy: At beginning, PLWD may still be able to be involved in medication-use process and maintain autonomy Desire to respect autonomy by involving in decisions and medication management More difficult to respect autonomy as dementia progresses (ethical dilemma vs. optimal medication use) Supervision of medication taking may become necessary
Scheduling and administration of medications	Dosing schedules and multiple medications may be problematic Change in medications require support to incorporate in schedule Daily routine affected by dosing schedule Develop individualised approach to administer medications	Disruptions to round affect medication timing Organisation of medication round and medications need improvement to make it efficient Reviews needed to de-prescribe to make round shorter More staff may help with limiting distractions	Increase in number and variety of medication and complexity of regimen: Contributes to caregiver stress and burden Affects PLWD’s willingness to take medications
Choice of formulation	Unsuitable formulations may cause PLWD and caregivers to use inappropriately or resort to incorrect modifications, leading to exacerbation of PLWD’s health PLWD and their caregivers have individual preferences relating to size, colour, taste, and ease of use that need to be taken into account when prescribing Choice of formulation important to ensure medication taken by PLWD	Physical impairments (swallowing, eyesight) should be considered Residents may have individual preferences and can refuse medications Route of administration making formulations unsuitable and/or treatment failure Unsuitability of formulations result in modification → safety and efficacy issues Need better prescribing decisions to ensure medications are suitable and able to be taken safely Reviews needed to assess suitability of route of administration and formulation	Development of swallowing difficulties: Chewing solid oral dosage form (SODF) Increase in swallowing difficulties Modification of medication or switch in formulations (SODF to liquids) Appropriateness of formulations: Difficulties with inhalers, pessaries, eye drops, etc. Formulations and route of administration may become unsuitable and require change
Interactions with and between providers	Ineffective communication and loss of continuity may result in caregivers and PLWD not voicing medication concerns Caregivers and PLWD should be included in consultations to provide full scope of medication-use issues and resolve them accordingly Pharmacist great source of medication information, especially regarding alternative formulations and should endeavour to ask caregivers and PLWD about medication use	Better communication between the MDT and care staff to address issues appropriately and support care-home staff Potential role of pharmacist within care home to optimise medication use	Support needs change along trajectory depending on dementia severity and caregiver health More active partnership between PLWD/caregiver and healthcare providers (HCPs) with shared decisions in consultations
Information and knowledge	Lack of knowledge affects medication taking and decisions surrounding medication use Information needed on appropriateness of medications, side effects, effectiveness, interactions, modifications, and need Lack of medication information to make informed decisions may contribute to caregiver burden	Lack of staff knowledge on medication modification implications Need to know how to recognise swallowing difficulties to take appropriate measures concerning medications Need training on administering various formulations and how to deal with challenging behaviour	Signpost support services and appropriate sources of information along progression for PLWD and their caregivers HCPs should acknowledge changes in information needs as disease progresses, such as alternative formulations, correct modification of medications if required, how to identify side effects, effectiveness, and need for medications.

**Table 4:** Consultation tool.

How are the PLWD and their caregiver managing with the number of medications and dosing schedule, especially alongside the caregiver’s medications and daily routine?
Are there issues encountered with swallowing certain formulations (tablets, capsules, liquids), or is the PLWD chewing medications before swallowing?
Acceptability of the taste, size, and shape of formulations?
If the caregiver has ever broken a tablet or opened a capsule and why?
Explain the occurrence of swallowing difficulties and the need to alert the GP if they occur.
How are the PLWD and caregiver using/administering the inhaler and how are they fitting it in their schedule (assess inhaler technique)?
Assessing the PLWD’s autonomy: ○ How involved is the PLWD with medication use? ○ Does the caregiver explain what the medications are for? ○ Do the caregiver and PLWD share responsibilities? ○ How are decisions regarding medications made? (Such as giving PRN medications or when the PLWD refuses a dose)

## Data Availability

The data presented in this study are available on request from the corresponding author.
